# The aging-disease false dichotomy: understanding senescence as pathology

**DOI:** 10.3389/fgene.2015.00212

**Published:** 2015-06-16

**Authors:** David Gems

**Affiliations:** Institute of Healthy Ageing and Department of Genetics, Evolution and Environment, University College LondonLondon, UK

**Keywords:** aging, evolution, disease, pathology, senescence

## Abstract

From a biological perspective aging (senescence) appears to be a form of complex disease syndrome, though this is not the traditional view. This essay aims to foster a realistic understanding of aging by scrutinizing ideas old and new. The conceptual division between aging-related diseases and an underlying, non-pathological aging process underpins various erroneous traditional ideas about aging. Among biogerontologists, another likely error involves the aspiration to treat the entire aging process, which recent advances suggest is somewhat utopian. It also risks neglecting a more modest but realizable goal: to develop preventative treatments that partially protect against aging.

## Introduction

Is our understanding of aging still in the dark ages? Over the course of the last centuries a gradual process of enlightenment has taken place in many different areas of human understanding, in which traditional views have been overturned by new knowledge borne of reason and the results of scientific investigation. A more realistic view of things, though it can initially cause controversy by upsetting traditional views and practices, ultimately enables more effective and more ethical action. Such a process of rationalization has profoundly affected the field of medicine, and the way we view many health-related issues, such as surgery, hygiene, infection, vaccination, abortion, contraception, homosexuality, and many others.

Yet when it comes to aging this salutary process of rationalization is still in its early stages. Here a salient example is the widespread and, arguably, false view that aging is distinct from disease and therefore not appropriate for medical attention – and even something benign and wholesome (Kass, 1983; Callahan, 1994; Fukuyama, 2002). In this essay I will discuss the distinction drawn between aging and disease, starting with an account (in the form of a mild parody) of various traditional but largely false ideas about aging, some of them supported by this misunderstanding. I will then describe how its elimination yields a clearer picture of the greatest cause of human illness and death.

## Aging vs. Disease: A View from Tradition

According to traditional views, aging is part of the natural order of things that one should not resist. Although aging seems frightening at first, and it is tragic that we all have to die, in the end it is for the good. From the Judeo–Christian perspective, it is our just punishment for Original Sin, specifically that of Eve, who ate the fruit of the tree of knowledge of good and evil – *cherchez la femme*. Aging also serves a function in nature: to weed out old and worn out individuals thereby freeing up resources for younger generations. In this way, aging assures the survival of the species. Thus, one should endure aging and bow out gracefully with stoicism and dignity in accordance with nature’s wishes. Conversely, to refuse to accept aging is a sign of weakness of character, of egotism, like a rich man who tries to avoid paying his taxes – and, of course, it is folly. There is a right length of life: 3 score and 10 years (i.e., 70); to want more is unseemly, greedy, and selfish.

Aging is not a disease, but rather a normal and natural process. Older people do tend to get ill more often and to develop serious diseases such as cancer and Alzheimer’s disease. Thus, although aging itself is not a disease, it is associated with an increased burden of disease. Treating these diseases is the duty of doctors, and finding new treatments for them a major priority of medical research. Such research is worlds apart from the folly of trying to resist aging.

There are scientists that try to understand the biological basis of aging. Their priority should be to understand how aging gives rise to age-related disease in order to find ways to protect older people from late-life illness. The aim of this research is not to slow aging or increase lifespan, but to increase *healthspan*, and *to add life to years, not years to life*. The ultimate outcome of biogerontology would be treatments that liberate older people entirely from pathology, yet allow them to age naturally. This would allow each of us to then eventually die of *pure aging*, feeling well one day, and then dying suddenly the next – perhaps in our sleep. However, this outcome would not be unproblematic, as it would cause an increase in the fearful anticipation of death among the elderly. As expressed by philosopher Leon Kass: “Would not the fear and loathing of death increase, in the absence of its antecedent harbingers?” And “Death would always be untimely, unprepared for, shocking” (Kass, 1988). Perhaps, therefore, it might be better after all if older people were left with some age-related disease, some suffering, at least near the end of life, to help ease the prospect of inevitable death.

Another concern is that some scientists studying aging pander to that egoistic minority who wish to live longer than their allotted time. These scientists openly advocate going beyond looking for treatments for diseases of aging, and intervening in aging itself. Disturbingly, they have managed to achieve this using animals models in the laboratory. Some even claim that aging itself is a disease. This betrays a contempt for older people, and promotes their marginalization (Haber, 2004). It also represents inappropriate medicalization of the elderly – as if they did not have enough to suffer already. Intervening in aging itself could have horrible consequences, as foreseen by the ancient Greek myth of Tithonus, the lover of Eos Goddess of the Dawn. To humor her matrimonial aspirations, Zeus bestowed immortality upon Tithonus – but not eternal youth. Consequently, poor Tithonus grew ever more decrepit but, cruelly, could not die. It would seem that some scientists studying aging are irresponsible, lacking in human feeling, and do not know when to stop.

## Aging is Disease: An Enlightened Perspective

The previous section collects together views that I have encountered many times during my 20 years working as a biogerontologist, often from members of the public but also from clinicians, gerontologists, and academics of various other specialities. In the main these ideas are, I believe, quite false. A particular source of error is the false dichotomy drawn between aging and disease.

To lay bare this error, a good place to start is an examination of the word *aging*, which can create confusion because it has several different meanings. For one, it can mean an increase in calendar age; likewise the phrase *old age* usually means advanced calendar age (though in the phrase *dying of old age* it means more than that). Then it can mean changes that occur with increased calendar age (*age changes*). In living organisms age changes usually refer to those that happen during adulthood, not before. Age changes in adulthood include those that are beneficial, such as the hardening of the cuticle in young adult insects, continued growth in trees, and experience in people. These are benign *maturational* changes, and *maturity* is a virtue. Age changes can also be deleterious, for example in aging *Caenorhabditis elegans*, the worm that I study in my own research, one sees many pathologies, including breakdown of muscle, intestinal atrophy, and uterine tumors (Garigan et al., 2002; Herndon et al., 2002; Luo et al., 2010); in humans, such changes are very numerous and diverse, ranging from cardiovascular and neurodegenerative disease to osteoporosis and cataracts. Such deteriorative changes exemplify *senescence* which, as defined by Alex Comfort, results in “a decrease in viability and an increase in vulnerability” and “an increasing probability of death with increasing chronological age” (Comfort, 1979). Like other nouns with the suffix *–escence*, which can indicate either a state, or a process of becoming, senescence can mean either the state of senescence, or the process of becoming senescent (c.f. obsolescence). When biogerontologists speak of aging they usually mean senescence (though not when speaking of successful or healthy aging). Thus, the fallacy that is the focus of this essay is, more precisely, the senescence-disease false dichotomy.

Is senescence a disease? The very word *senescence*, denoting deterioration leading to death, certainly carries that implication. To explore this, the philosopher Arthur Caplan asked two further questions: Does aging have a purpose? And: Is it distinct from pathology? (Caplan, 1992, 2005).

If senescence is an evolutionary adaptation, this would to some extent support the idea that aging is non-pathological. But this reasoning would also involve a fallacious appeal to nature, a false equation of human evolutionary fitness with well being. If human aging did in fact evolve to benefit the species by ridding it of worn out elderly people, this should not deter us from looking for treatments for Alzheimer’s disease and cancer.

However, as Caplan emphasizes, this 19th century evolutionary theory of aging has been largely superceded by another, developed by J.B.S. Haldane, Peter Medawar and George C. Williams in the mid-20th century. According to the predominant contemporary theory, aging is not an adaptation in any sense (Haldane, 1941; Medawar, 1952; Williams, 1957); although not all biogerontologists adhere to this view (Longo et al., 2005). That it did evolve reflects the fact that evolutionary fitness is ultimately a function of reproductive success, not of individual long-term survival; and also pleiotropy, the capacity of a gene mutation to affect different traits in different cell types and organs and at different times. Because of pleiotropy, genetic variants can appear in populations that cause changes in early life that enhance reproductive success, but in later life lead to increased pathology. But, critically, the disease and death that such *antagonistic pleiotropy* causes in later life has little effect on evolutionary fitness. This is because mortality in the wild from extrinsic causes (e.g., from predation, starvation, and disease) mean that few individuals survive long enough to develop senescent pathologies (Williams, 1957). To be precise, the pleiotropic effects proposed by Williams (1957) may not necessarily be antagonistic, in so far as the impact of late life pathologies on fitness may be negligible.

Thus, evolutionary theory tells us that aging is something very much like a genetic disease: it is a set of pathologies resulting from the action of pleiotropic gene mutations. Yet it is not quite the same. Consider conventional genetic disease mutations, such as those causing Duchenne muscular dystrophy (DMD), affecting the dystrophin gene. Such mutations are wholly deleterious, such that tackling DMD could involve correcting the deficiency pharmacologically or with gene therapy. But in aging, antagonistic pleiotropy means that the same genes that give us life cause our death. For example, target of rapamycin (Tor), a strong candidate for a gene that is subject to antagonistic pleiotropy, promotes both development to adulthood, and the development of many late-life pathologies (Blagosklonny, 2010). Yet as in a conventional genetic disease, the option exists to correct the deleterious effects of such aging genes – but in later life, after the early beneficial effects have been expressed. Consistent with this, giving the Tor-inhibitory drug rapamycin to middle-aged mice can extend their lifespan (Harrison et al., 2009).

Coming to Caplan’s other question: is aging distinguishable from pathology? Given the similarity in meaning between *disease* and *pathology*, asking this is similar to asking whether aging is a disease. This also touches on another interesting question, namely: in biological terms, what exactly constitutes pathology? A full pathobiological account of a disease provides an explanation of the origins and causes of disease and illness in terms of biological malfunction. For diseases as conventionally defined, there is often a clear correspondence between biological malfunction and disease; for example, infection with the influenza virus can cause severe illness. But biological malfunctions often occur that do not produce illness, and so are not identified as pathology. For example, freckles are caused by sun damage to melanocytes (I have freckles; a nurse in Egypt once asked me: “Do they hurt?”); I also enjoy a glass of whisky from time to time, which is a form of intoxicant. Some senescent changes (e.g., skin wrinkling) fall into this category of pathobiology without illness, this gray area between health and sickness. The problem of precisely defining terms such as disease and pathology are the subject of much philosophical debate, which lies beyond the scope of this essay.

An account of the biological mechanisms that cause aging could help to answer the question of whether this process is pathological: are its underlying causes recognizably a form of biological deterioration, or something else? Here a snag is that our understanding of the biology of aging remains embryonic, though much progress has been made in recent decades (Blagosklonny, 2008; Kenyon, 2010; Bartke et al., 2013; Campisi, 2013; Vijg and Suh, 2013). The evolutionary theory of aging implies that antagonistic pleiotropy is a cause of aging, but does not identify the actual biological processes that antagonistic pleiotropy affects.

Numerous factors have been identified which play a role in aging, such as cellular senescence, DNA damage and inflammation, reviewed in (López-Otín et al., 2013). Biogerontologists have also sought broader generalizations about mechanisms of aging. For example, a long-standing theory is that aging results from the accumulation of molecular damage caused, for example, by reactive oxygen species (ROS) such as the superoxide ($O_{2}^{•-}$) free radical (Harman, 1956; Sohal and Weindruch, 1996; Kirkwood and Austad, 2000). According to this view, cellular maintenance processes that protect against damage also protect against aging. If molecular damage causes aging then this implies a fundamentally pathological process.

However, in recent years negative results of tests of the ROS theory have raised doubts about how important molecular damage is as a cause of aging; reviewed by (Van Raamsdonk and Hekimi, 2010). This has led some to think outside of the damage-maintenance box altogether and to consider alternative paradigms (e.g., de Magalhães, 2012; Gladyshev, 2013). In particular, it was proposed by Blagosklonny (2006, 2008) that the primary cause of aging is not damage and loss of function, but rather too high activity (or *hyperfunction*) of genes, and pathways in later life. Blagosklonny’s (2006, 2008) ideas are strongly rooted in Williams’s concept of antagonistic pleiotropy, but also incorporate recent findings from experimental biogerontology, e.g., studies of lifespan genetics, dietary restriction, and cellular senescence. But like the earlier damage-maintenance paradigm (and the concept of antagonistic pleiotropy), the hyperfunction theory implies that aging is fundamentally a pathological process.

As Caplan observes, the mechanisms proposed to cause aging are similar to those known to underlie many disease processes. He concludes that there is no clear distinction between aging and pathology (Caplan, 1992, 2005), and this is supported by accumulating evidence from biology. Having spent many years studying aging in worms, the age changes one observes involve accumulating pathology – nothing else. One way to define aging (i.e., senescence) in biological terms is as the set of pathologies that increase with advancing age; most of these pathologies have endogenous origins, although they can be strongly influenced by environmental factors, and many (but not all) senescent pathologies can contribute to mortality (Gems, 2014); see also (Kulminski et al., 2007; Rockwood and Mitnitski, 2007). Consistent with this definition, treatments that extend lifespan in animal models typically delay age-related pathology and extend youth span: life extension only occurs as the result of prevention of pathology, whether the pathology is caused by aging or by something else. Life extending treatments in the laboratory invariably decelerate aging (rather than stopping or reversing it); this results in a delay in the appearance of age-related pathology (extending youthspan), but then such pathologies still eventually appear, causing illness, and death.

With an understanding that aging is pathology, many of the notions in the previous section lose validity. For example, the goal of preventing diseases of aging without altering aging itself makes little sense if aging itself is pathological, though it certainly makes sense to prioritize action against the more lethal pathologies. Also Kass’s creepy scenario of vigorous, healthy elderly people fearfully facing imminent death appears highly improbable – thankfully.

**FIGURE 1:**
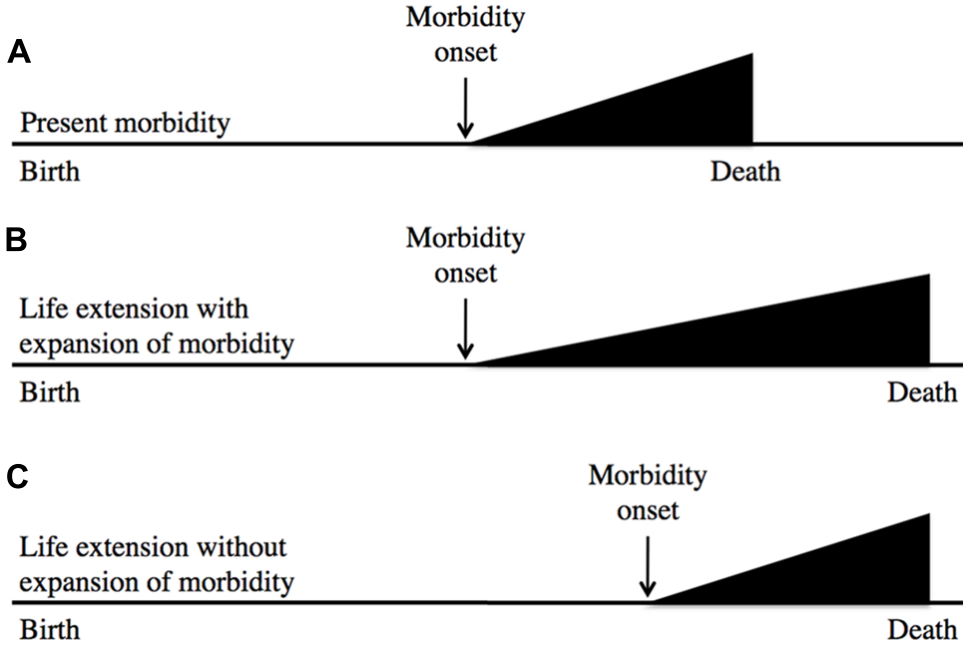
**Geroprotection is likely to reduce relative lifetime morbidity.** This represents the age increase in morbidity either without **(A)** or with **(B,C)** life extension. After Fries et al. (2011), which suggests **(B)** as a likely outcome of life extension. Model organism studies, and rejection of the aging-disease dichotomy imply that **(C)** is more likely. Here the horizontal axis represents time. In relative terms, **(C)** involves a compression of morbidity.

In a similar vein, the likelihood of recapitulating Tithonus’s dreadful fate is very remote; in fact, to my knowledge no biogerontologist has ever generated a worm or fruitfly Tithonus in which life in a state of advanced senescence is greatly extended. A more plausible concern is that geroprotective treatments that increase lifespan could lead to expansion of morbidity (i.e., an increase in the proportion of life spent in ill health due to aging; Fries et al., 2011). This is possible, but assessment of the worth of such treatments must also include the likely benefits in terms of expansion of the healthy period of life. Based on animal model studies a plausible outcome of geroprotection is a compression of lifetime morbidity in relative terms (i.e., as a percentage of time alive), but not in absolute terms (i.e., years of morbidity; Blagosklonny, 2012); this is described using a standard representation in **Figure 1**

Finally, the goal of enabling people to die without pathology, or of pure aging, is untenable if non-pathological senescence does not exist. In fact, the idea of elderly people dying of aging without pathology is plainly nonsense, as noted previously (Blagosklonny, 2006); among the defining properties of pathology, causing death is surely a *sine qua non*. (Yet I recently discussed with a former director of a major medical research funding agency the idea that elderly people can die without pathology, and found that they agreed with it).

It seems likely that advances in biogerontology will contribute to geroprotective interventions which hold back the pathologies of human aging; such interventions may well increase lifespan. A recurrent feature of arguments against treating aging is an over-emphasis on increased lifespan as an outcome, and neglect of alleviation of illness. Thus, to say: “I would like a longer life” may be presented as egoism or folly, but not “I would like to remain free of cancer.” Likewise, one would not hold against someone infected with, say, malaria their wish not to die from the disease – and one would certainly not accuse them of egotism for wishing to extend their life. The point here is that, in the end, senescence is in many ways just like other severe diseases: it causes illness and death, and treating it results in a longer life. Critics of treating aging are often guilty of double standards, and of undervaluing the well being – and life – of older people.

## Biogerontological Utopianism and the Definition of Geroprotection

Even among biogerontologists opinions differ about whether aging is a disease; for a recent argument against see (Rattan, 2014). But there is another idea about aging that is current among biogerontologists that I believe impedes biogerontology from benefiting medicine, which involves the notion of a central process of aging.

A major point of reference for biogerontologists is the fact that a number of interventions can cause large extensions in both healthspan and lifespan in animal models (Kenyon, 2010; Bartke et al., 2013). This is most striking in *C. elegans*, where up to 10-fold increases in adult lifespan have been achieved (Ayyadevara et al., 2008). One interpretation of such findings is that there exists a central process of aging that causes all age-related pathology. If one could intervene in this process in humans one might achieve protection against the full spectrum of age-related diseases (Holliday, 1996; Butler et al., 2008). Thus, an aspiration of biogerontology has been to develop geroprotective therapies that intervene in this central aging process – in aging itself, thereby achieving great benefits in terms of protection against late-life disease.

This is a beautiful and inspiring prospect, yet one may find fault with it, in several respects. First, it relies on the assumption that there exists such a central aging process. While the damage-maintenance paradigm could imply that there is one, the hyperfunction theory does not. The former suggests that augmentation of somatic maintenance could slow the entire aging process – even dramatically, leading to large increases in lifespan. The disposable soma theory predicts that in terms of evolutionary fitness “the optimal level of investment in somatic maintenance […] is less than the level required for indefinite survival,” i.e., that increased somatic maintenance could prevent aging altogether (Kirkwood and Rose, 1991). A further deduction drawn is that aging may be reversible (de Grey et al., 2002).

By contrast, the hyperfunction theory does not predict the existence of a unitary, central aging process. Instead it suggests that lifespan is limited by a set of pathologies caused by evolved levels of activity of, for example, the Tor and insulin/IGF-1 signaling (IIS) pathways that are too high for optimal function in later life (Blagosklonny, 2006, 2008). The fact that inhibition of Tor/IIS increases lifespan does not signify that its over-activity causes the entire aging process. That lifespan is not extended indefinitely by reducing Tor/IIS could reflect newly life-limiting pathologies of different etiologies (e.g., due to hyperfunction in other pathways, or perhaps to stochastic molecular damage; Blagosklonny, 2008).

**FIGURE 2:**
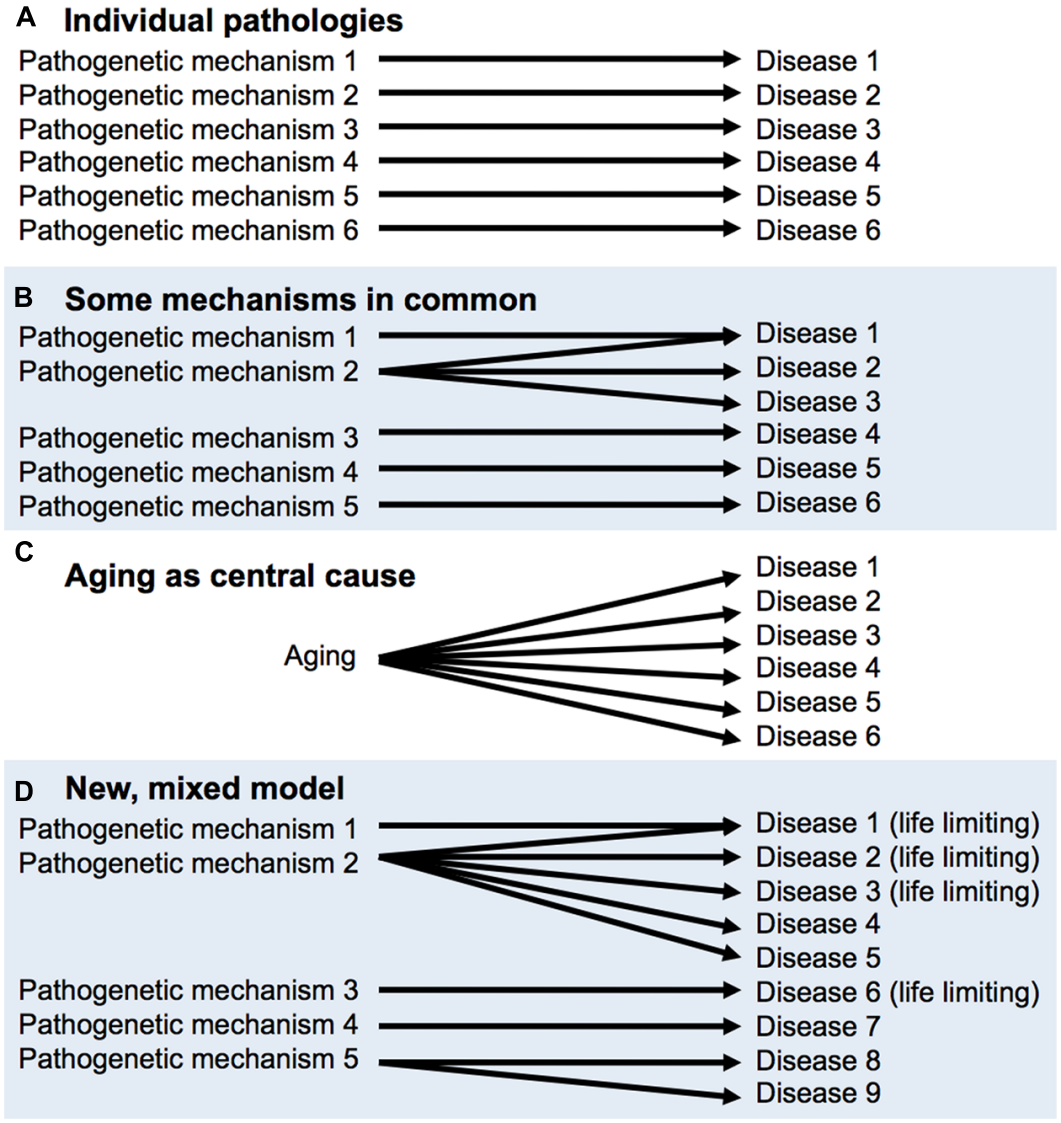
**FIGURE 2. Aging as a cause of age-related disease: different models. (A,B),** derived from Peto and Doll (1997). **(A)** There is no aging process, only individual diseases of aging. **(B)** A few diseases of aging may have shared mechanisms, **(C)** A central aging process causes diseases of aging; the utopian view. **(D)** Mixed model, based on recent findings and concepts. Some pathways (e.g., target of rapamycin/insulin/IGF-1 signaling, Tor/IIS) promote many pathologies, including a number that limit healthspan and lifespan. But this does not mean that Tor/IIS controls the aging process, only part of it.

This latter view is reconcilable with the declaration of eminent medical researchers Peto and Doll (1997) that “There is no such thing as aging,” meaning that there is no single process underlying aging. One possible hypothesis about senescence is that it is a collection of individual pathologies, each which has its own time-dependent, cumulative etiology, e.g., mutation accumulation for cancer, β-amyloid aggregation for Alzheimer’s disease and so on. If this were correct, it would make little sense to study aging overall. However, Peto and Doll also opine that “some [age-related phenomena] will probably have part or all of their mechanisms of origin in common, but some may not.” Critically, the discovery of the effects on late-life health and lifespan of Tor/IIS and other pathways demonstrates that more aging pathologies have common mechanisms than had previously been supposed; for a diagrammatic representation of this, see **Figure 2** But this does not mean that Tor/IIS controls *the* aging process, that causes all diseases of aging. An argument against the utility of the individual pathologies view (**Figure 2**) is that treatment of one age-related pathology can sometimes exacerbate another (Barzilai, 2014).

The second issue is that such utopianism neglects a lesson from biogerontology of particular utility in terms of achieving improvements in late life health: the efficacy of *preventative* approaches to decelerate the development of age-related pathologies. This is as true of interventions that prevent many pathologies and considerably extend lifespan (as in the effects of dietary restriction on rodents) as of interventions that suppress a smaller number of, or individual pathologies.

The question of how to define anti-aging (or geroprotective) treatments is an open one, and I have discussed it more fully elsewhere (Gems, 2014). Arguably, it is both more realistic and more useful to employ the broader definition of geroprotection, that includes protection against multiple or single pathologies of aging. Effective deployment of narrow-spectrum geroprotectants is something that is more achievable in the short term, and would help to establish a practice and tradition of geroprotection that could over time be extended to the use of broader spectrum geroprotectants. As defined, some effective narrow-spectrum geroprotectants already exist. One example is the regular use of sun-block to prevent the slow accumulation of solar damage to the skin. Another, that is currently being tested, is the use of the cardiovascular polypill to reduce risk of cardiovascular disease (Wald and Law, 2003). I suspect that the more utopian definition of geroprotection, though inspiring to biogerontologists, is a turn-off for clinicians, who view it as unrealistic.

## Conclusion

To act ethically a realistic grasp of relevant facts is critical. This is particularly important for aging, the main cause of chronic disease and death in the world today. Yet traditional ideas about aging include some major misconceptions, including the aging-disease false dichotomy. It is to be hoped that such ideas do not misguide those responsible for the healthcare interests of older people, including those responsible for setting medical research priorities. Neglect resulting from misunderstanding aging may cause harm by allowing preventable illness, both now and in the future – given that geroprotection is most efficacious in the form of prevention. To achieve the best outcomes in terms of the future health of older people, it is vital to adopt a frank and rational attitude to aging. We must draw aside the rosy veil of tradition and face aging for what it is, and in all its horror: the greatest disease of them all.

## Conflict of Interest Statement

The author declares that the research was conducted in the absence of any commercial or financial relationships that could be construed as a potential conflict of interest.
